# Check the Ear. The Importance of Ear Examinations in Assessment of Intracranial Subdural Empyema

**DOI:** 10.3390/tropicalmed4030120

**Published:** 2019-09-18

**Authors:** Joseph Yoon, Michael Redmond

**Affiliations:** Department of Neurosurgery, Royal Darwin Hospital, Darwin Northern Territory, Tiwi 0810, Australia

**Keywords:** foreign body, otitis media, otitis externa, intracranial subdural empyema, indigenous, fungal infection

## Abstract

Intracranial subdural empyema (ISE) is an uncommon condition previously associated with almost 100% morbidity and mortality. Since the introduction of antibiotics and advancements in diagnosis the complication rates have significantly improved. We report an unusual case of a 32-year-old Aboriginal male diagnosed with ISE. On closer inspection the ISE was found to be a complication of otitis media with a cotton bud lodged in the external acoustic meatus. The report provides a literature review on the relationships of ISE, otitis media and foreign bodies. We conclude that although rare, all patients with suspected ISE should undergo an ear examination as it is at no cost to the patient or health service but may be the difference between life and death.

## 1. Introduction

Before the advent of antibiotics, intracranial subdural empyema (ISE) had mortality reaching nearly 100% within 24–48 h from presentation [1,2,3,4,5,6,7]. The treatment of precursor infections with antibiotics has significant reduced the rates of complications in ISE to 15%–41% [2,3,4]. Further improvements in investigative techniques has reduced time to diagnosis further decreasing complications to 6–15% [1]. ISE is no longer a death sentence with mortality now < 4% [5,7,8]. We report a case of a 32-year-old Aboriginal male with left sided sub-temporal ISEs secondary to otitis media and externa. Intraoperatively we found the presence of a foreign body resembling a cotton bud within the external acoustic meatus abutting the tympanic membrane. To our knowledge this is the second reported case of ISE complicated by otitis media with a foreign body. We review the literature of ISE, otitis media and highlight the importance of clinical suspicion and ear examination in patients presenting with features of ISE. 

## 2. Case

**Figure 1:**
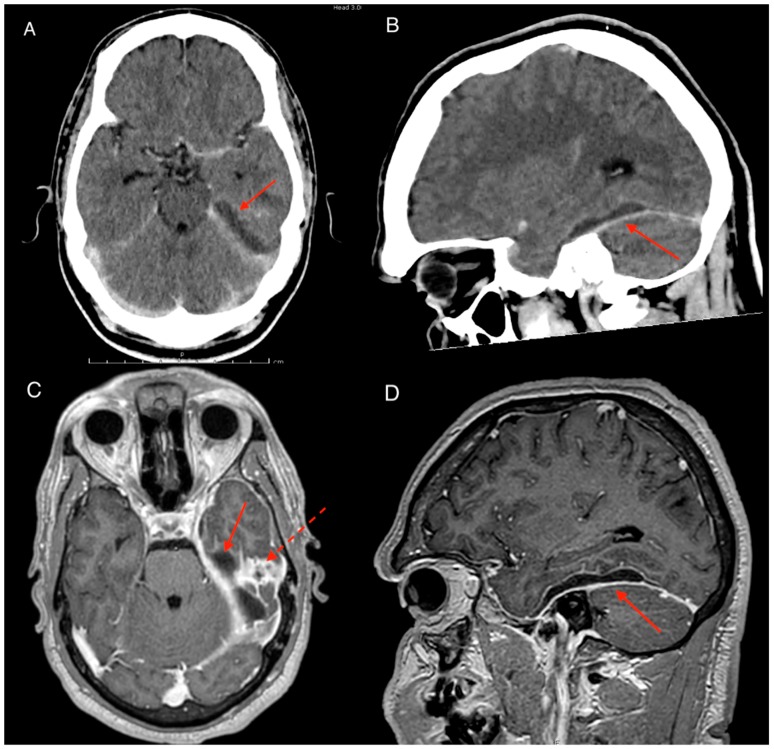
Imaging on presentation, contrast computerized topography (CT) and magnetic resonance imaging (MRI) brain T1 contrast axial and sagittal views. (**A**) Axial CT scan with contrast showing subdural collection in the left middle cranial fossa (red arrow). (**B** & **D**) Sagittal CT with contrast and MRI T1 with contrast showing collection above the tentorium. Note minimal contrast enhancement of the collection with no clear evidence of cerebral oedema representing early stages of intracranial subdural empyema (ISE). (**C**) Axial MRI T1 with contrast. Note area of contrast enhancement at the floor of middle cranial fossa on the petrous part of temporal bone (dotted arrow) correlated with the dural defect found intraoperatively.

A 32-year-old Aboriginal male presented to the local community doctor 100 km from Darwin NT Australia with a 1-week history of severe headaches and vomiting. On presentation the patient had fevers of 40.2 degrees Celsius but otherwise had normal vital signs. He was expediently transferred to our service via air ambulance. Patient had a background history of cognitive impairment and stage 2 chronic kidney disease. He was a non-smoker with moderate intake of alcohol in the community. Clinical examination showed no neurological deficits with Glasgow coma scale (GCS) of 14/15 with 1 point lost in verbal test due to baseline communication disability. Blood tests revealed white cell count (WCC) of 19.9 × 10^9^/L (neutrophilia) and c-reactive protein (CRP) count of 213 mg/L. Blood cultures were also taken which returned negative. The combination of symptoms and suspicion of an intracranial infection prompted a computerized topography (CT) brain scan which revealed an extra-axial collection with rim enhancement. The collection was located at the floor of the left middle cranial fossa with associated vasogenic edema of the surrounding brain suggestive of a subdural empyema (Figure 1A,B). Closer inspection on CT of the ear canal revealed opacification of the middle ear, mesotympanum and epitympanum with consistent findings on the magnetic resonance imaging (MRI) (Figure 1C,D). During the MRI the patient experienced seizures and was loaded with levetiracetam and placed on regular doses.

**Figure 2:**
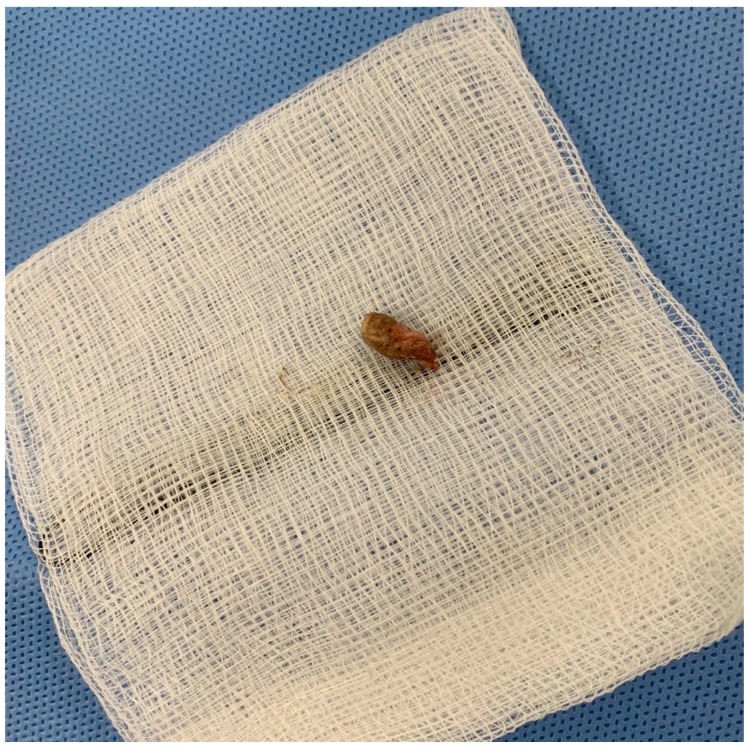
Foreign body found in the left ear approximately 1 cm × 0.5 cm × 0.5 cm on gauze. Firm to touch with fibrous material.

The patient was started on empiric antibiotic therapy for subdural empyema with vancomycin, meropenem and amphotericin. Due to the location of the empyema and suspicion raised from the CT scan our team requested the ear, nose and throat (ENT) team for their opinion. The ENT team attempted visualisation of the ear canal but noted difficulty in identifying the tympanic membrane and opted for closer inspection and examination under anaesthesia. There was a delay in surgical intervention due to lack of clinical resources requiring more time for surgical planning. During this time the patient deteriorated with re-emergence of fevers and early signs of haemodynamic instability. This prompted emergency sub-temporal craniotomy on day 4 of admission with the ENT team also present for exam under anaesthesia with consideration of myringotomy and insertion of grommets. On incision of the dura, copious amounts of frank purulent discharge were encountered. The temporal lobe herniated through the cranial and dural defect requiring mannitol and lowering of the blood pressure. After adequate medical management, the temporal lobe was retracted away from the tentorium and liberal amounts of wash was used till clear fluid returned. We noted a dural defect at the floor of the middle cranial fossa approximately 5 mm in diameter. This was patched with a pericranium graft and overlayed with collagen matrix. Due to herniation and concern of gross contamination of the bone flap, a decision was made to close without bone in-situ. Post-closure of the cranial wound, the second stage of the procedure was continued by the ENT team. Under the microscope the patient showed signs of otitis externa with acoustic meatus completely occluded by diffuse erythema and swelling approximately 2 cm from the canal opening. Careful retraction of the canal wall revealed a 1 cm × 0.5 cm × 0.5 cm foreign body resembling a cotton bud (Figure 2). The material was removed using micropituitary, and the ear canal irrigated. A grommet was not inserted at this time due to complete occlusion of canal and the surgery concluded with plans to review patient again after adequate time to allow swelling to settle. Intraoperative swabs grew *pseudomonas aeruginosa*, *streptococcus constellatus* and *scedosporium apispermum*. Antibiotics were rationalised from sensitivities and the patient was placed on meropenum and voriconazole.

**Figure 3:**
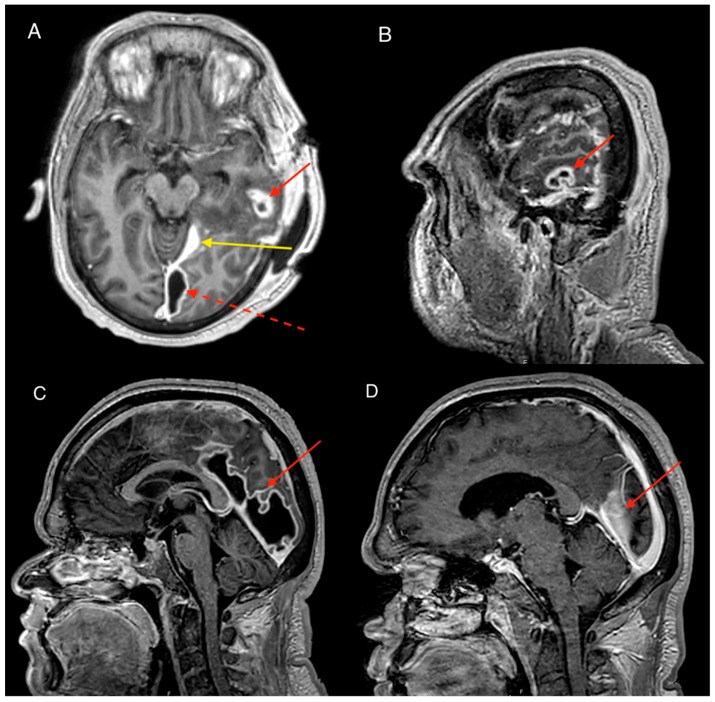
MRI brain with contrast. (A & B) Axial and sagittal scans after first surgery illustrating development of intracerebral abscess (red arrow) and development of parafalcine collection (dotted arrow). Note dural thickening and enhancement of the tentorium (yellow arrow). (C & D) Comparison MRI pre-operative to second surgery and then 4 weeks later showing complete resolution of collection in the parafalcine area.

Post operatively, the patient had significant clinical improvement including normalisation of vital signs and blood markers with return to his previous cognitive baseline. A CT scan on post-operative day 1 however revealed a new para-falcine collection located at the left occipital region. Although the patient was well clinically, resolving inflammatory markers plateaued on post-operative day 6 with CRP in the range of 60–100 mg/L and WCC of 14–16 × 10^9^/L. A repeat MRI revealed the para-falcine collection had enlarged with increase in the thickness of the collection wall with new development of intracerebral abscess within the left temporal lobe (Figure 3A–C). After discussion between the infectious disease and neurosurgical team a consensus was reached for occipital craniotomy with re-opening of the previous sub-temporal craniotomy. Intraoperatively, copious amounts of purulent discharge were drained from the new occipital craniotomy, and swabs taken. Dural biopsy was done at the same time which confirmed *Scedosporium apiospermum* growth which was previously considered possible contaminant. After the second procedure the patient made a marked recovery with both normal vital signs and inflammatory markers of CRP < 5 mg/L and WCC of 7.5 × 10^9^/L within the post-operative week. The ENT team reviewed the patient again under the microscope showing significant improvement of the swelling of the external acoustic meatus. The tympanic membrane was perforated at this time, and free discharge of pus was noted. The decision was made to not perform grommet insertion, in favor of free drainage. Prior to discharge into the community *Hospital in the Home* program, concerns were raised by clinical staff regarding the possibility of foreign material in patient’s ear. On closer investigation the patient and his father revealed that they had attempted to plug the ear to prevent purulent discharge ruining his clothes and sheets. Education was provided to the family and a loose gauze was applied over the ear for patient comfort. The patient was followed up at 1 month and had no further stigmata of infection with repeat MRI showing no identifiable collection (Figure 3D). Further outpatient reviews at 2 and 3 months revealed no concerns of infection.

## 3. Discussion

### 3.1. Epidemiology

The clinical epidemiology of ISE is greatly affected by the geographical location and the period of time the studies were published. Most studies show ISE predominantly affects children and young adults with 71% of patients aged between 6–20 years of age with a slight male predominance. In these studies the majority of ISE are associated with meningitis, sinusitis and otogenic diseases [2,3,4,6,8,9]. Compared to these studies, French et al (2014) recently published a retrospective analysis of a 10-year case series in Australia with contrasting results. Their paper showed ISE to be more prevalent in the older population with a mean age of 46.1 years. It also showed the source of infection was majorly attributed to neurosurgical procedures (44%) followed by sinusitis (28%) and otogenic disease (14%). Although the difference in the results may be a reflection of the low study number, the study may highlight the importance of social and cultural paradigms in determining risk of ISE. We hypothesize the younger, male population that was seen in the earlier studies were at an increased risk of ISE due to their parallel higher risk of sinusitis, meningitis, dental caries and trauma [4,9]. 

Clinical epidemiology of ISE secondary to otogenic infection show a younger population with mean age of 26.9 years (6 months–79 years) with again a slight male predominance of 58% [3,10]. Otitis media itself causes intracranial complications with an incidence of 1.97%–4.18% [10]. A majority of otitis media associated intracranial complications are meningitis however a smaller portion develop intracranial abscesses [2,11]. Taken together the risk of developing ISE from chronic otitis media is <0.1% [3,10]. 

Otitis media remains a large public health concern in the Aboriginal and Torres Strait Islander population. Compared to their non-indigenous counterparts, otitis media occurs earlier, persists longer and presents as more clinically severe [12]. The prevalence of otitis media in Aboriginal and Torres Strait Islanders has been shown to reach 30%–39% at 24 months of age [13]. The range of active chronic otitis media was shown to range from 10.5% to 30% [12,13]. The prevalence is even higher in remote areas than urban communities [12]. These figures are in excess of the 4% prevalence which the World Health Organisation specifies as a significant public health problem [12,14]. The intricacy of the health gap between the Indigenous and Non-Indigenous population is complex and out of the scope of this paper but reflects disadvantage as a root of these issues. Some socio-economic factors worth mentioning include but not limited to adverse living conditions, poor infrastructures and domestic overcrowding which can lead to increased rates of bacterial colonisation in this population. These factors compounded by smoking and poorer health hygiene multiplies the risk of developing otitis media [12,15,16]. Public health schemes such as pneumococcal vaccinations, chlorinated pools, and public awareness campaigns have unfortunately shown no change in the prevalence of otitis media in the Aboriginal and Torres Strait Islander population in the last 30 years [12]. Ultimately, the health gap remains a complex problem and despite being located in Australia, the Aboriginal and Torres Strait Islander populations resemble risk profiles of developing populations [13]. This raises the importance of social history in our clinical assessment and without prejudice should assist in our assessment of patients. 

### 3.2. Pathogenesis

Intracranial complications from otitis media occur when the suppurative process of the ear accesses the central nervous system. In patients with ISE and otitis media, cholesteatoma was present in as many as 78% of patients with chronic otitis media and 18% of acute otitis media [2,3,10]. Another mechanism of entry is through direct invasion by osteomyelitis. Intracranial complications are commonly located in the temporal lobe and cerebellum which highlights the direct route of otitis media into the intracranial compartment [2,3]. In our case, access into the subdural compartment was illustrated by the dural defect overlying the temporal bone which has also been previously reported [8]. This localized process may explain the low yield in blood cultures in ISE with positive results, in only 5% of cases [5,8]. It is hence recommended all patients undergo intraoperative swabs which can remain sterile in up to 18% of cases [17]. Another route of infection into the intracranial compartment is inoculation through bacteremia and septic embolization of small vessels of the dura, venous sinuses and endolymphatic channels [3,11,17]. The presence of the inflammatory mass in the subdural compartment with irritation and mass effect is responsible for the clinical symptoms. In adults 81% experience headaches, 75% fevers, 67% altered sensorium, and 50% of patients experience vomiting [5,8]. The clinical triad of fevers, headaches and vomiting in ISE is present in half of patients [1,3,5,17]. The mean duration of symptoms was 15 days but can be as early as 2 days and as late as 6 weeks [1,5,8,18].

Although there is an established association between foreign bodies and otitis media, its role in the disease process is not clear [19]. The foreign body may have caused otitis media/externa but also may be inserted as a consequence of otitis media/externa related irritation. Regardless, the presence of a foreign body could act as a continuous source of infection and accelerate the disease and render antibiotic therapy obsolete if not removed. In our case this was exemplified by the erythema and associated swelling of the ear canal adjacent to the foreign body. To date, we can only identify one previous study with a foreign body associated otitis media causing intracranial abscess [7]. Goldman et al (1998) reported a 51-year-old lady diagnosed with otitis media whom interestingly continued to insert foreign materials into the ear, much like our patient. In our case, the patient inserted the foreign body the second time as a means to prevent discharge overflowing out of his ear. More recently Charlton, Janjua and Rejali (2019) reported a case of a 31-year-old male with necrotizing otitis externa secondary to a cotton bud with a CT brain revealing ISE [20]. In this case the foreign body is likely the cause of ISE but in the earlier case seems to reflect a consequence of ISE. Irrespective, we believe the presence of a foreign body may act to accelerate otitis infections and increase the risk of intracranial complications. We therefore recommend that all patients with suspicion of ISE undergo ear examination and prompt imaging. CT is the initial recommended modality but scans can be normal in up to 63% which reflect early stages of disease [4,18,21]. If there is still a strong level of concern for intracranial infection than an MRI should be ordered. MRI has greater sensitivity of 93% and concurrently a magnetic resonance venogram can be done to assess for sinus thrombosis [18]. Of note, more than half of patients have more than one intracranial complication on diagnosis [3,10].

### 3.3. Management 

Once diagnosis is established, the patient should be promptly treated with broad spectrum antibiotics [2,8]. Combinations such as third generation cephalosporin with metronidazole and vancomycin to cover streptococcus species and staphylococcus aureus are recommended [22]. In our cohort due to the relatively large incidence of atypical infections such as melioidosis and fungal species, patients are treated with a combination of vancomycin, meropenem and amphotericin until cultures return. Typically antibiotics are given over a period of 6 weeks, however depending on source control and clinical outcome this may continue indefinitely [8,17,22].

Depending on the presentation, management by lowering the intracranial pressure may be required including administration of mannitol, elevating the head of the bed and maintaining normocapnia [1,22]. Prophylactic anticonvulsants are recommended due to the high rates of seizures associated with ISE [1,17,23]. Conservative management alone however is not recommended unless the patient has a small collection with significant improvement with antibiotics early in admission [24]. As seen in our case a delay in surgical treatment can lead to rapid expansion of the ISE highlighting the lethal and morbid nature of this condition.

Most patients should undergo prompt surgical washout of the subdural empyema which can be done by craniotomy or burr holes. Previously, burr holes were not considered an acceptable form of therapy. However there have been studies showing no difference in outcomes in treatment with burrholes compared to craniotomy [22,25,26]. In our opinion, the decision on management will depend on the patient, the abscess and surgical expertise. In situations where patients are not well enough for a large craniotomy, the collection is thin, not loculated or there are limitations to surgical expertise, then a prompt burr hole may be a lifesaving and morbidity limiting procedure. On the other hand, if there is an extensive collection with thick loculations with adequate surgical support then a craniotomy is recommended. Our technique of choice is a craniotomy. This allows for assessment of loculated collections, removal of membranes and adequate washout of the collection. A burr hole would have been technically difficult in our case to clear the collection under the temporal lobe. Our approach also allowed us to identify the dural defect that was likely to be the entry point of the infection into the subdural space and allowed us to patch this defect. 

The decision to remove or replace the bone flap is under contention. If there is evidence of contamination of the bone such as established osteomyelitis then removal of the bone may yield favourable results [17,26]. In our case, the herniation on dural opening made the decision for us. The treatment of otitis media should also be done preferentially before or concomitantly with neurosurgical intervention [3]. In cases of cholesteatoma, an open technique was the treatment of choice with the closed technique reserved for cases of non cholesteatomatous chronic otitis media [3,27,28].

## 4. Conclusions

The presence of a foreign body in the external auditory canal can act as a nidus for infection and lead to devastating complications such as an intracranial subdural empyema. Although extremely rare, we stress the importance of ear examination in cases of suspected ISE as a means to promptly identify and implement treatment. It is a relatively quick and easy exam with negligible cost or risk to the patient. Our case also illustrates the importance of early antibiotics and urgent surgical intervention. We believe the delay in surgical management likely caused extension of the infection, placing the patient at a high risk of complications and also the need for a repeat surgical procedure. In essence our team recommends high levels of suspicion, to start treatment early, and to remember to always check the ear when approaching patients with intracranial subdural empyema.
